# Assessing stability and change of four performance measures: a longitudinal study evaluating outcome following total hip and knee arthroplasty

**DOI:** 10.1186/1471-2474-6-3

**Published:** 2005-01-28

**Authors:** Deborah M Kennedy, Paul W Stratford, Jean Wessel, Jeffrey D Gollish, Dianne Penney

**Affiliations:** 1 School of Rehabilitation Science, McMaster University, Hamilton, ON, Canada; 2 Centre for Studies of Physical Function, Orthopaedic and Arthritic Institute of Sunnybrook and Women's College Health Sciences Centre. Department of Physical Therapy, University of Toronto, Toronto, ON, Canada; 3 Department of Clinical Epidemiology and Biostatistics, McMaster University, Hamilton, ON, Canada; 4 Division of Orthopaedic Surgery, Orthopaedic and Arthritic Institute of Sunnybrook and Women's College Health Sciences Centre. Department of Surgery, Faculty of Medicine, University of Toronto, Toronto, ON, Canada

## Abstract

**Background:**

Physical performance measures play an important role in the measurement of outcome in patients undergoing hip and knee arthroplasty. However, many of the commonly used measures lack information on their psychometric properties in this population. The purposes of this study were to examine the reliability and sensitivity to change of the six minute walk test (6MWT), timed up and go test (TUG), stair measure (ST), and a fast self-paced walk test (SPWT) in patients with hip or knee osteoarthritis (OA) who subsequently underwent total joint arthroplasty.

**Methods:**

A sample of convenience of 150 eligible patients, part of an ongoing, larger observational study, was selected. This included 69 subjects who had a diagnosis of hip OA and 81 diagnosed with knee OA with an overall mean age of 63.7 ± 10.7 years. Test-retest reliability, using Shrout and Fleiss Type 2,1 intraclass correlations (ICCs), was assessed preoperatively in a sub-sample of 21 patients at 3 time points during the waiting period prior to surgery. Error associated with the measures' scores and the minimal detectable change at the 90% confidence level was determined. A construct validation process was applied to evaluate the measures' abilities to detect deterioration and improvement at two different time points post-operatively. The standardized response mean (SRM) was used to quantify change for all measures for the two change intervals. Bootstrapping was used to estimate the 95% confidence intervals (CI) for the SRMs.

**Results:**

The ICCs (95% CI) were as follows: 6MWT 0.94 (0.88,0.98), TUG 0.75 (0.51, 0.89), ST 0.90 (0.79, 0.96), and the SPWT 0.91 (0.81, 0.97). Standardized response means varied from .79 to 1.98, being greatest for the ST and 6MWT over the studied time intervals.

**Conclusions:**

The test-retest estimates of the 6MWT, ST, and the SPWT met the requisite standards for making decisions at the individual patient level. All measures were responsive to detecting deterioration and improvement in the early postoperative period.

## Background

Osteoarthritis, the most common reason for total hip (THA) and knee arthroplasty (TKA), accounts for more difficulty with climbing stairs and walking than any other disease [1,2]. Physical performance measures, therefore, play an important role in the measurement of outcome in patients undergoing total joint arthroplasty. Although the past two decades have seen considerable development and evaluation of self-report functional status measures [3-7] these advances have not been paralleled to the same extent in performance measures.

Information about customary or normal values often exists for performances measures, however, information concerning sensitivity to change and clinically important change are rarely available [8]. This gap is exemplified in the case of commonly used performance measures in the assessment of patients post TKA and THA. Measures such as self-paced walk tests (SPWTs) [9-11], the timed up and go test (TUG) [9,12,13], stair measures (STs) [9-11,14] and the six minute walk test (6MWT) [14-18] lack information on responsiveness in this population [8]. Although the literature contains varied definitions of responsiveness, in this case, it is used to indicate the ability of a measure to detect change [19].

A few studies have examined the responsiveness of the 6MWT and STs in patients following arthroplasty. Kreibich et al [15] investigated the responsiveness of six outcome measures using paired t tests and found that the 6MWT was more responsive than a thirty-second stair climb, yet not as responsive as the two disease specific measures studied. Parent et al [14] compared the responsiveness of 3 locomotor tests and 2 questionnaires using 4 different responsiveness statistics and recommended the 6MWT and the Physical Function subscale of the Western Ontario and McMaster Universities Osteoarthritis Index (WOMAC) for assessment in the early recovery period after TKA. No studies were found that examined the responsiveness of the SPWT and TUG. Several studies used performance test components in other tools, however, they were not reported in their original format [20,21].

Responsiveness statistics such as the standardized response mean (SRM) and effect size (ES) are important for making relative comparisons between measures. However, clinicians still require estimates to quantify the error in patients' scores and to determine if change has truly occurred. In the absence of population specific benchmarks, clinicians and researchers apply the results available from other populations. For example, Mahon et al [17] used the 6MWT as one outcome measure to examine the association between waiting time and postoperative health-related quality of life in patients undergoing THA. They considered a change of greater than 30 meters in the 6MWT to be clinically important, based on the work of Guyatt et al [22] in respiratory patients. Enhancing the interpretability of commonly used performance measures in the end stage OA-arthroplasty population would assist clinicians and researchers to better quantify decline and recovery.

The importance of determining THA and TKA population specific benchmarks is further underlined when one considers the growing number of North Americans requiring total joint arthroplasty [23,24]. In Canada alone, the number of THR and TKR increased 31.7% from 1994/1995 to 1999/2000 [25]. The purposes of this study were therefore to examine the reliability and sensitivity to change of the SPWT, TUG, ST and the 6MWT in patients with end-stage hip or knee osteoarthritis (OA) who subsequently underwent a total joint arthroplasty.

## Methods

### Subjects

The sample consisted of patients with a diagnosis of OA who were scheduled to undergo primary, unilateral THA or TKA and was part of a larger, observational, longitudinal study. A sample of convenience was chosen and included one hundred fifty consecutive, eligible patients (69 hips, 81 knees) investigated over the one-year period, November 2001 to 2002. Eligibility criteria included the following: diagnosis of OA, scheduled for primary total joint arthroplasty; sufficient language skills to communicate in written and spoken English; and absence of neurological, cardiac, psychiatric disorders or other medical conditions that would significantly compromise physical function. Patients were excluded if they were scheduled for revision, bilateral or staged arthroplasties. All of the surgeries took place at a specialized, orthopaedic tertiary care hospital in Toronto.

The characteristics of the patients with respect to age, height, weight, and body mass index (BMI) are reported in Table 1. All patients provided informed consent and the study received approval from the institution's research and ethics review board.

**Table 1:** Sample Characteristics

n = 150	Mean, SD	Quartiles
Age (yr)	63.7, 10.7	57, 64, 72
Height (m)	1.69, 0.09	1.62, 1.68, 1.76
Weight (kg)	85.5, 15.4	74.3, 83.3, 94.2
Body mass index (kg/m^2^)	30.0, 4.9	26.3, 28.9, 33.4

n, Number of subjects

Yr, year

M, meter

Kg, kilogram

SD, standard deviation

### Outcome measures

As noted earlier, patients completed four timed performance measures; the fast SPWT, TUG, ST, and 6MWT, at each assessment point. Time was measured on a stopwatch to the nearest 1/100 of a second. The order of testing was as follows: SPWT, TUG, ST, and 6MWT with a 10 minute rest between the ST and 6MWT. Standardized guidelines for performing the SPWT, TUG, and ST have been reported previously for a similar patient population [9,11]. In terms of the fast SPWT, patients were timed while they walked two lengths (turn excluded) of a 20-m indoor course in response to the instruction: "walk as quickly as you can without overexerting yourself." The ST required patients to ascend and descend 9 stairs (step height, 20 cm) in their usual manner, and at a safe and comfortable pace. To complete the TUG, patients were required to rise from a standard arm chair, walk at a safe and comfortable pace to a tape mark 3-m away, then return to a sitting position in the chair [26]. During the performance of the 6MWT, patients were instructed to cover as much distance as possible during the 6 minute time frame with opportunity to stop and rest if required. The test was conducted on a pre-measured, 46 meter unobstructed, uncarpeted, rectangular circuit. The course was marked off in meters and the distance traveled by each subject was measured to the nearest meter. As encouragement has been shown to improve performance [27], standardized encouragement, "You are doing well, keep up the good work" was provided at 60 second intervals. During the administration of each of the four performance measures, patients were permitted to use their regular walking aids.

### Study design

**Figure 1:**
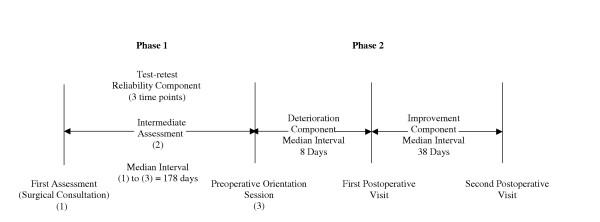
Study Design

As noted previously, the data for this study represent a subset from a larger ongoing study that examines recovery profiles using a number of self-report and physical performance measures. The study has two arms, in Phase 1 patients are recruited from the caseload of two orthopaedic surgeons with high volumes and long waiting lists to examine the impact of waiting time on recovery profiles. In phase 2, patients are recruited from all of the orthopaedic surgeons' lists at their preoperative visit to the hospital's standardized patient orientation program, which is scheduled one to two weeks prior to surgery. There are no differences in the postoperative follow-up for both of the Phases and all patients receive standardized treatment, following either a primary total hip or knee care pathway. To provide an accurate model of change over time, patients' follow-up measurements are scheduled at different intervals. The format is that of an observational repeated measures' design (Figure 1).

Test-retest reliability was assessed preoperatively in a sub-sample of 21 patients from Phase 1. These 21 patients represented individuals who had progressed to surgery and follow-up by the time of this analysis. Data from patients' initial consultations with the surgeon, an intermediate assessment, and then again at patients' preoperative orientation visits contributed to the reliability analysis. Although the median interval between the first and second assessments was 91 days (1^st^, 3^rd ^quartiles: 72, 133 days) and between the first and third assessments was 178 days (1^st^, 3^rd ^quartiles: 140, 204 days), there is evidence to suggest that the amount of change in function while on the waiting list is minimal [28]. A second strategy was also employed to examine the stability of the twenty-one patients' measures over the aforementioned time period using data from the larger study on the Lower Extremity Functional Scale (LEFS). Previous research has determined the LEFS minimal detectable change at a 90% confidence level (MDC_90_) to be 9 LEFS points [29]. Using this benchmark, data from only 17 of the 21 patients were retained for the reliability analysis.

It is important when assessing responsiveness that a research design be employed in a period where change is expected. Based on the results of prior work [9], it was recognized that the early period following joint arthroplasty would provide such a framework in which the measures' abilities to detect deterioration and improvement could be determined. A construct validation process was therefore applied to evaluate the measures' abilities to detect change at two different time points post-operatively. The first postoperative assessment occurred within 15 days of surgery. The median interval between the preoperative and first postoperative assessment was 8 days (1^st^, 3^rd ^quartiles: 7, 9 days). It was theorized that patients' lower extremity functional status, as represented by either the time to complete a task or the distance covered in the case of the 6-minute walk test, would demonstrate deterioration compared to their preoperative values [9]. Next it was theorized that patients' lower extremity functional status would improve over the interval between the first and second postoperative assessments with the minimum interval between these assessments set to 20 days. The median interval between these postoperative assessments was 38 days (1^st^, 3^rd ^quartiles: 32, 46 days).

### Analysis

Descriptive statistics including the mean, standard deviation, and quartiles were applied to summarize the data. Shrout and Fleiss Type 2,1 intraclass correlation coefficients (ICC) were used to describe the measures' test-retest reliabilities [30]. Standard errors of measurement (SEMs) were used to quantify the measurement error in the same units as the original measurement [31]. The 95% confidence intervals for all ICCs and SEMs [30,31] were calculated. In addition, the error associated with a measured value (i.e., 90% confidence interval) and the minimal detectable change at the 90% confidence level (MDC_90_) was calculated [19]. The error calculation for a measured value was obtained by multiplying the point estimate for the SEM by the z-value associated with the 90% confidence interval (z = 1.65). To calculate MDC_90_, the value obtained from the error calculation was multiplied by the square root of two (i.e. MDC_90 _= SEM × 1.65 × ). The interpretation of MDC_90 _is that 90% of truly stable patients will demonstrate random variation of less than this magnitude when assessed on multiple occasions. A change greater than MDC_90 _is often interpreted as a true change.

The standardized response mean (SRM) was used to quantify change [3] and SRMs were calculated for all measures for the two change intervals. A minus sign was applied to all SRMs that represented deterioration in functional status. For example, a decrease in distance, and an increase in time were assigned negative values. Although sample values of the SRM for the measures represent estimates of the population parameters for these measures, it is impossible to directly ascertain their sampling distributions. We applied a bootstrap procedure to obtain approximate representations of the sampling distributions for the measures' SRMs and to estimate their 95% confidence intervals [32]. Bootstrapping involves sampling with replacement. Specifically, 1000 samples of size n – where n equaled the number of observations for the specific analysis of interest – were selected with replacement. Estimates of SRMs were ordered from lowest to highest; accordingly, the 25^th ^and 975^th ^observations from the bootstrap samples represented the 95% confidence limits. This method provides a distribution free estimate of the confidence limits.

## Results

**Figure 2:**
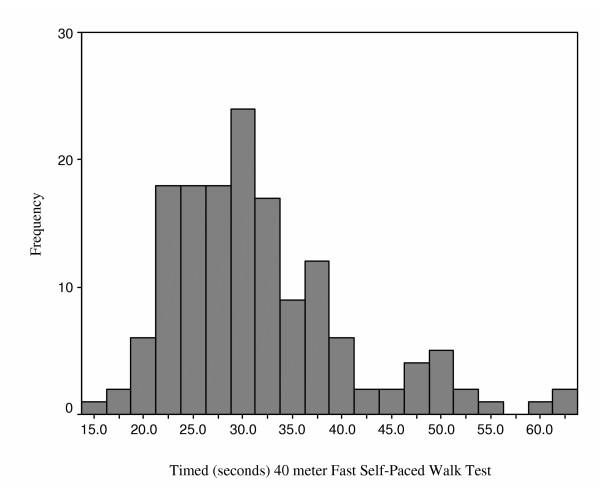
Distribution of Times to Complete the Fast Self-Paced Walk Test

**Figure 3:**
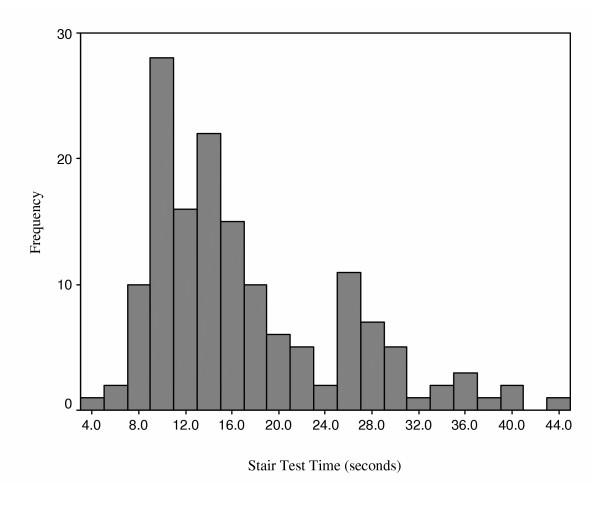
Distribution of Preoperative Stair Test Times

**Figure 4:**
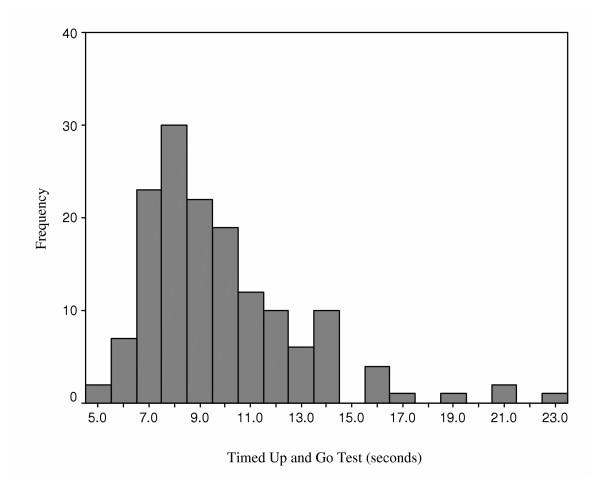
Distribution of Preoperative Timed Up and Go Test Times

**Figure 5:**
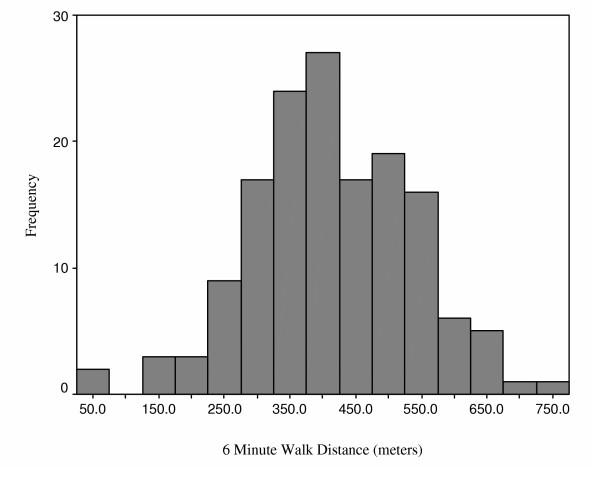
Distribution of Preoperative 6 Minute Walk Test Distances

Figures 2, 3, 4, 5 provide the distributions of preoperative scores for each of the performance measures. Table 2 provides a summary of the reliability analyses and estimates of SEM and MDC_90_. There was no systematic difference between the test and retest assessments for any of the measures (p > 0.05). All of the estimates were greater or equal to 0.90 with the exception of the TUG. Table 3 summarizes the measured performance values (means and quartiles) for the three assessment points and Table 4 presents a summary of the change scores and SRMs. The number of patients in Tables 3 and 4 differ as a result of the pattern of missing values. The results presented in Tables 3 and 4 provide consistent evidence that lower extremity functional status, as represented by the time/distance concept, deteriorates between the preoperative and first postoperative assessment. The measures demonstrated uniform improvement from the first to second postoperative assessments: time decreased, and distance for the 6-minute walk increased. As apparent in Table 4, the SRMs were greatest for the ST and 6MWT over the two measured time intervals. Table 5 provides an accounting of the missing data. It is evident from this table that a substantial number of patients were unable to complete the ST and 6MWT when administered within 16 days of surgery. Independent t-tests were performed to test if the preoperative values differed for patients who were and were not able to complete the ST and 6MWT at the first postoperative visit. No significant differences (p > 0.05) in the preoperative ST or 6MWT were observed for patients in the two groups.

**Table 2:** Reliability Coefficients and Minimal Level of Detectable Change

Measure	R (95% CI)	SEM (95% CI)	Confidence in Score (90% CI)	MDC_90_
Fast Self-paced Walk Time (completed over 40 meters)	0.91 (0.81, 0.97)	1.73 (1.39, 2.29)	± 2.86 s	4.04 s
Stair Time	0.90 (0.79, 0.96)	2.35 (1.89, 3.10)	± 3.88 s	5.49 s
Timed Up and Go Time	0.75 (0.51, 0.89)	1.07 (0.86, 1.41)	± 1.76s	2.49 s
Six Minute Walk Test Distance	0.94 (0.88, 0.98)	26.29 (21.14, 34.77)	± 43.37 m	61.34 m

R, Reliability Coefficient

SEM, Standard Error of Measurement

MDC_90_, Minimal detectable change at the 90% confidence Level

s, seconds

m, meters

**Table 3:** Mean and Quartile Scores of the Performance Measures across Time

Measure	Preop Mean, SD Quartiles n = 150	Postop 1 <16 Days Postop Mean, SD, n Quartiles	Postop 2 >20 Days From Postop 1 Mean, SD, n Quartiles
Self-paced Walk Time (seconds)	31.7, 9.2 25, 30, 36	85.7, 62.7, 115 53, 66, 93	33.7, 10.9, 92 26, 32, 38
Stair Time (seconds)	17.1, 8.2 11, 15, 22	40, 12, 87 29, 39, 48	20.0, 9.7, 91 12, 18, 27
Timed Up and Go Time (seconds)	9.8, 3.2 7, 9, 11	24.7, 14.2, 116 15, 21, 31	10.3, 4.2, 91 7, 9, 12
Six minute Walk Test Distance (meters)	412, 123 329, 412, 508	193, 87, 82 120, 194, 263	408, 116, 91 328, 393, 477

SD, Standard Deviation

n, Number of subjects

**Table 4:** Change Scores and Standardized Response Means

Measure	Preop to First Postop Interval Mean Change*, SD, n SRM* (95% CI)	First to Second Postop Interval Mean Change*, SD, n SRM* (95% CI)
Self-paced Walk Time (seconds)	-54.8, 61.6, 115 -0.89 (-1.42, -0.68)	47.7, 60.7, 89 0.79 (0.66, 1.45)
Stair Time (seconds)	-23.8, 13.8, 87 -1.74 (-2.13, -1.45)	20.59, 10.40, 73 1.98 (1.68, 2.42)
Timed Up and Go Time (seconds)	-14.9, 13.8, 116 -1.08 (-1.38, -0.92)	13.57, 13.04, 89 1.04 (0.84, 1.61)
Six minute Walk Test Distance (meters)	-232, 133, 82 -1.74 (1.60, 1.97)	207, 109, 61 1.90 (1.46, 2.39)

* Negative sign indicates a worsening in the measured value; positive sign indicates an improvement in the measured value

SD, Standard Deviation

SRM, Standardized Response Mean

n, Number of subjects

CI, Confidence Intervals

**Table 5:** Missing Values Details

Measure	Time 2 Eligible n = 119	Time 3 Eligible n = 93
		
Self-paced Walk		
Completed Test	115	92
Unable to Complete Test	4	0
Missing	0	1

		
Stair Test		
Completed Test	87	91
Unable to Complete Test	29	1
Missing	3	1

		
Timed Up and Go Test		
Completed Test	116	91
Unable to Complete Test	3	0
Missing	0	2

		
Six minute Walk Test		
Completed Test	82	87
Unable to Complete Test	33	1
Missing	4	5

## Discussion

This study has provided information concerning the measurement properties of four performance measures used to complement information concerning lower extremity functional status in patients with advanced OA undergoing THA or TKA. The test-retest reliability component of this study was conducted over a median interval of 178 days, which is a longer period than would typically be chosen to assess stability. This extended reassessment interval was chosen to accommodate the fact that random measurement error is often time dependent, and in practice, the period between clinical visits is often greater than several months [33]. A potential concern when applying a reassessment of this duration is that true change in the sample will occur; however, in this study the LEFS MDC_90 _was applied to further define a stable patient sample. The reliability coefficients (Table 2) for the time and distance components of the tests met or exceeded 0.90 with the exception of the TUG. They are believed to represent conservative estimates of the reliability likely to be associated with most clinical reassessment intervals.

It is important to remember that the reliability of a measure intended for individual patient application must be greater than the reliability of a measure designed for group use [34]. Different authors have advocated different standards for individual patient use, Nunnally [34] recommended 0.95, Kelley [35] 0.94 and Weiner and Stewart suggested 0.85 [36]. Although the reliability of the TUG at 0.75 would meet the standards for group application, it would not meet the aforementioned standards for individual patient use. The SPWT, ST and 6MWT would meet one or all of these standards.

In reviewing the mean and quartile scores of the performance measures preoperatively (Table 3), the scores indicate higher function than those reported in other studies [14,16,17], including the findings from our own prior work which examined a large dataset of over 1800 patients [11]. One potential explanation for these findings may have been the age of our sample, 25% of the patients were 57 or younger. As noted in the Canadian Joint Replacement Registry, the numbers of THA and TKA in the 45–54 year age group has increased between 1994/1995 to 1999/2000 [25]. A second factor potentially accounting for the preoperative scores is the nature of the study. Individuals who could not complete all the performance measures preoperatively would not be included, thereby filtering out the individuals with the highest disability.

To be useful in clinical practice, the scores obtained on outcome measures must have meaning to clinicians. In this study, the SEM was used to identify the error associated with a patient's reported score and to estimate the value of MDC_90_. Because the SEM is reported in scale points, it enhances the interpretability of a patient's score and change score. To the authors' knowledge this is the first study to provide estimates for MDC_90 _for each of the four physical performance measures in the hip/knee end stage OA-arthroplasty population. These benchmarks will assist clinicians to more effectively monitor change in these types of patients.

Using a different methodology, Redelmeier et al [37] determined the smallest difference in the 6MWT associated with a noticeable difference in perceived walking ability for COPD patients to be a distance of 54 meters. Using this as a benchmark in arthroplasty patients would underestimate the distance required to be confident that a change had truly occurred. This illustrates the importance of population specificity when determining MDC_90_.

Many studies assessing change have focused on improvement only; the current investigation assessed deterioration and improvement [14,21,38,39]. Based on prior work, it was hypothesized that surgical intervention would induce a reduction in lower extremity functional status when assessed within 16 days of surgery [9]. All time/distance performance measures demonstrated deterioration over this interval. Subsequently all of the measures demonstrated significant improvements between the first and second postoperative visits. These findings suggest that the four performance measures are adept at assessing both types of change. The greatest changes were associated with the ST and 6MWT. Examination of the SRMs for these two tests demonstrated similar responsiveness over the studied time intervals.

This parallels the findings in the study by Parent et al [14] examining early recovery after TKA using locomotor tests, including gait speed, stair ascent cycle duration, and the 6MWT. Of these measures, the authors found the 6MWT to be most responsive over the study's three time points, ranging from preoperatively to 4 months postoperatively. Of interest, the stair ascent cycle duration, measured using a 2-dimensional biomechanical analysis system was least responsive and the authors recommended evaluating the responsiveness of a timed stair measure, which has been accomplished in this study.

In addition to providing information concerning the psychometric properties of the performance measures, our results also offer insights into the clinical application of these measures. The TUG was originally developed to easily evaluate the risk of falls using balance and basic functional mobility [8]. Tested in the frail elderly population, scores under 10 seconds were associated with individuals who were functionally independent [26]. Considering this benchmark and normative values reported for community dwelling elders [40], the patients' mean TUG score, in this sample, did not demonstrate much disability. Consequently, there would not be as much opportunity for detecting change. However, the usefulness of the TUG in an elderly orthopaedic population, including patients post THA and TKA, has been reported. [13].

In considering the SPWT and the 6MWT, it is not surprising that the 6MWT demonstrated greater responsiveness in this study, as it was measured over a longer distance and duration. Unlike the SPWT, which in this study was used to determine fast walking speed, the 6MWT has both speed and endurance components. However, as apparent in Table 5, the TUG and SPWT tests might be preferred if the goal was measurement in the early acute post-operative phase when patients deteriorate and may be unable to perform the ST or 6MWT. This was the case for over 25% of the current study's sample when assessed within 16 days of surgery. Therefore, the time period of administration and the patient's preoperative level of disability can serve as useful guides for clinicians faced with the decision of choosing the most informative measures.

This study has several limitations. As apparent in the tables, different numbers of patients were assessed at postoperative assessment one and two. This is partially a reflection of the study design, as mentioned earlier, not all patients were assessed at the same time points due to the goals of the larger ongoing observational study. However, some patients were also missed at both time points due to unexpected changes in appointments without communication to the investigators. Referral bias might also be a potential concern due to the nature of the institution being a specialized tertiary care facility. This must be balanced against the fact that it is one of the largest joint arthroplasty centers in Canada and draws from a wide catchment area. Considering the higher preoperative function of the patients in this sample, it will be important to replicate the current study's findings in different settings with other samples of arthroplasty patients. In addition, as responsiveness is a highly contextualized attribute [19], it would be informative to study the results over additional time points in the postoperative continuum.

## Conclusions

This study has examined selected psychometric properties in four commonly used performance measures to assess change in the end-stage OA-arthroplasty population. The test-retest reliability estimates of the SPWT, ST and 6MWT met the requisite standards for making decisions at the individual patient level. All of the measures were responsive to detecting deterioration and improvement in the early postoperative time period following arthroplasty. The time period of administration and the patient's preoperative level of disability can serve as useful guides for clinicians faced with the decision of choosing the most informative measures. Estimates of MDC_90 _have been reported for each of the performance measures to assist clinicians in assessing change.

## Competing interests

The author(s) declare that they have no competing interests.

## Authors' contributions

DMK conceived and designed the study, assisted with the statistical analysis and prepared the manuscript.

PWS assisted with the design, performed the statistical analysis and assisted with the manuscript preparation.

JW consulted in the conception and design of the study and assisted with the manuscript preparation.

JDG assisted with the design and execution of the study and manuscript preparation.

DP assisted in the coordination of the study, data collection and assisted with the manuscript preparation.

All authors read and approved the final manuscript.

## Pre-publication history

The pre-publication history for this paper can be accessed here:


